# The effect of long calcar screws on the primary stability of 3-part, varus impacted proximal humeral fractures compared to short calcar screws: a real fracture simulation study

**DOI:** 10.1007/s00402-022-04473-7

**Published:** 2022-05-30

**Authors:** Nadine Ott, Michael Hackl, Andreas Prescher, Martin Scaal, Fabian Lanzerath, Lars Peter Müller, Kilian Wegmann

**Affiliations:** 1 Department of Orthopedic and Trauma Surgery, Faculty of Medicine and University Hospital of Cologne, University of Cologne, Kerpener Street 62, 50937 Cologne, Germany; 2 Institute of Molecular and Cellular Anatomy -Prosektur, RWTH Aachen, Aachen, Germany; 3 Department of Anatomy, Faculty of Medicine, University of Cologne, Cologne, Germany; 4 Orthopädische Chirurgie München, OCM Clinic, Steinerstrasse 6, 81369 Munich, Germany

**Keywords:** Simulated humeral fractures, Screw, Calcar, Plate, Stability, 3-part, Length

## Abstract

**Background:**

Complex proximal humeral fracture ranks among the most common fracture types, especially in elderly patients. In locked plate fixation of proximal humerus fractures, the calcar is deciding for screws providing further medial column support. To date, the biomechanical effect of the length of these calcar screws is not well known. The purpose of this study was to analyze the effect of long calcar screws on fresh frozen prefractured cadaveric specimens.

**Methods:**

In the present biomechanical study, 8 pairs of cadaveric proximal humeri were fractured identically using a custom-made fracture simulator. ORIF was performed using a locking plate (PHILOS; Fa. Synthes). The specimens were tested in a biomechanical setup under increased axial load without any calcar screws installed, with short calcar screws and long calcar screws installed. Strain gages (4-wire-120 Ohm, Fa. Vishay) mounted on the locking plate were used to evaluate the fixation strain and to give an estimate for primary stability..

**Results:**

The measured strain of the locking plate without calcar screws (804,64 µm/m) at maximum load (200 N) was significantly higher than with short (619,07 µm/m; *p* = 0.02) or long calcar screws (527,31 µm/m; *p* = 0.007). Additionally, strain with short calcar screws was noticeably higher in comparison to long calcar screws (619,07 µm/m vs. 527,31 µm/m; *p* = 0.03).

**Conclusion:**

Use of calcar screws improves the stability of realistically impacted 3-part varus humeral fractures. Long calcar screws that are positioned as close as possible to the joint provide further primary stability compared to short calcar screws.

**Level of evidence:**

Basic science study.

## Introduction

Fractures of the proximal humerus are common upper extremity injuries, especially in elderly osteoporotic patients [1]. Due to the steadily increasing incidence of proximal humeral fractures in the elderly, the available treatment methods require further improvement. Open reduction and internal fixation (ORIF) is an established option to restore the joint and provide primary stability [2, 3]. However, fixation failure, humeral collapse with screw perforation may lead to poor clinical outcome in previous studies [3–5]. These complications are more severe in elderly patients and stable fixation is more difficult to achieve due to poor anchorage of screws in the osteoporotic bone [6].

Previous studies have focused on the use of calcar screws. These screws provide resistance to the humeral head collapse especially in varus impacted fractures [7, 8]. Although the role of calcar screws is not a novelty, to date, the effect of their length on primary stability is not well known. So, the aim of the present study is to evaluate stability of locked proximal humeral fractures and the related relevance of calcar screws. The presenting study is the first study using realistically simulated 3-part humeral fractures.

## Materials and methods

### Specimens

In the present biomechanical study, 8 pairs of fresh frozen cadaveric proximal humeri with a wide variety of bone mineral density were used.

The specimens were obtained from body donors; written consent was guaranteed. Institutional ethics committee approval was given prior to this study (VT 20 – 1002).

**Fig. 1:**
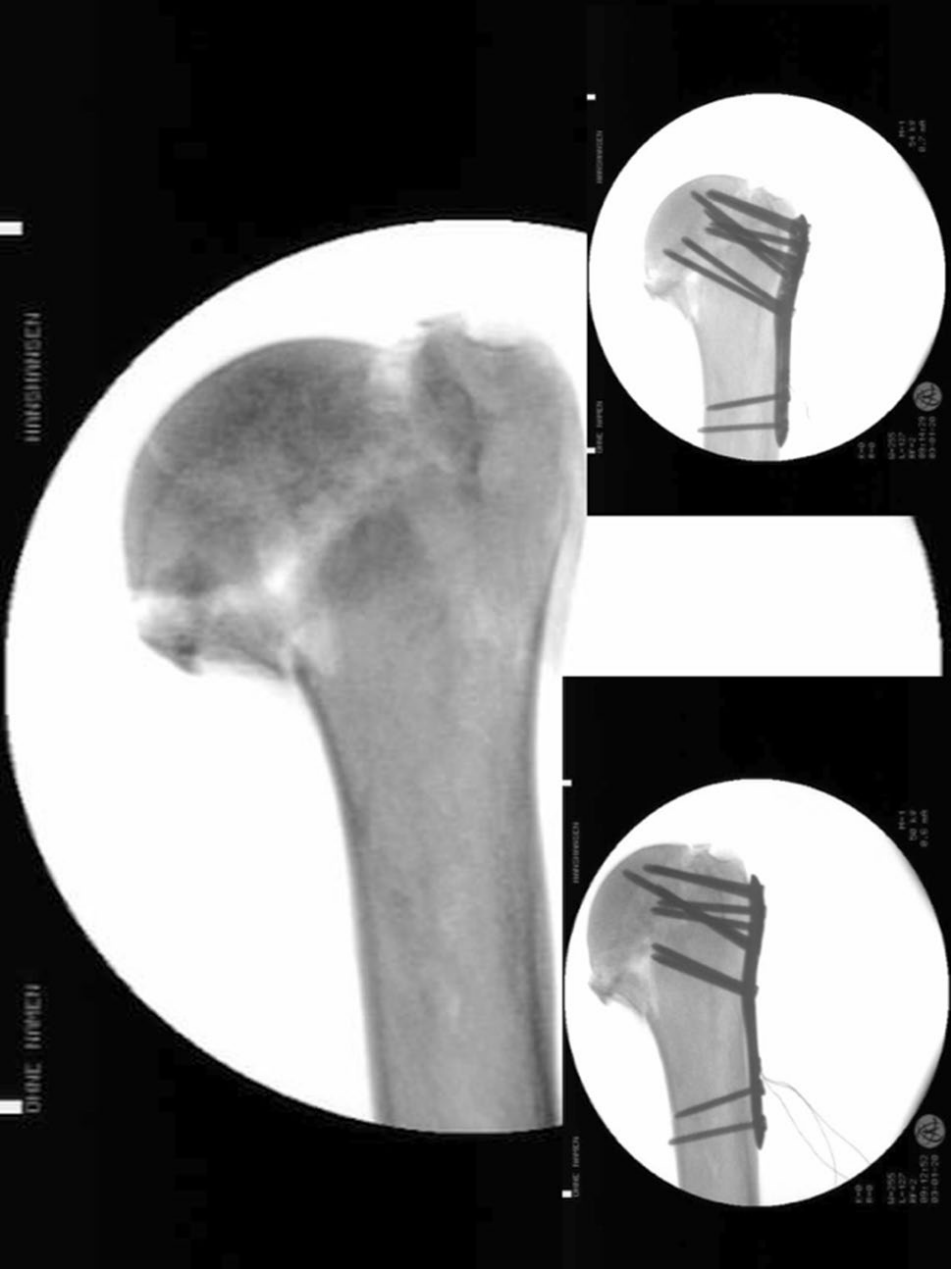
Radiographic scan (a.p.—view) of 3-part humeral fracture before and after locking plate with short and long calcar screws

The average age of the donors at the time of death was 80 years (min. 70; max. 93; SD 8.4), 4 donors were male and 4 were female. 8 right and 8 left specimens were available. Prior to testing, the specimens were radiographically controlled to detect any pre-existing pathology including fractures or osteoporosis, which would bias comparisons between the mechanical testing (Fig. 1). To analyze the bone density, CT scans were used and Hounsfield Units (HU) of the distal humerus (axial and sagittal) were measured.

The pairs were randomized to establish a control group, 8 out of 16 donor specimens received nor primary fracture and were tested in the biomechanical setup. The aim of the control group was to adjust and evaluate the biomechanical setup as well as the bending forces of the plate on non-fractured specimen.

### Test bench and fracture simulation

Prior to freezing at − 20 °C, all specimens were dissected free of any soft tissues. The specimens were thawed at room temperature 24 h ahead of testing. In the next step, the samples were grouted into custom-made steel squares using polymethyl methacrylate (PMMA). During the grouting process, a rectangular alignment of the humeri bone and the baseplate of the grouting area was ensured to allow precise axial force induction. Finally, the PMMA sample was fixed on a special baseplate in the test-bench.

**Fig. 2:**
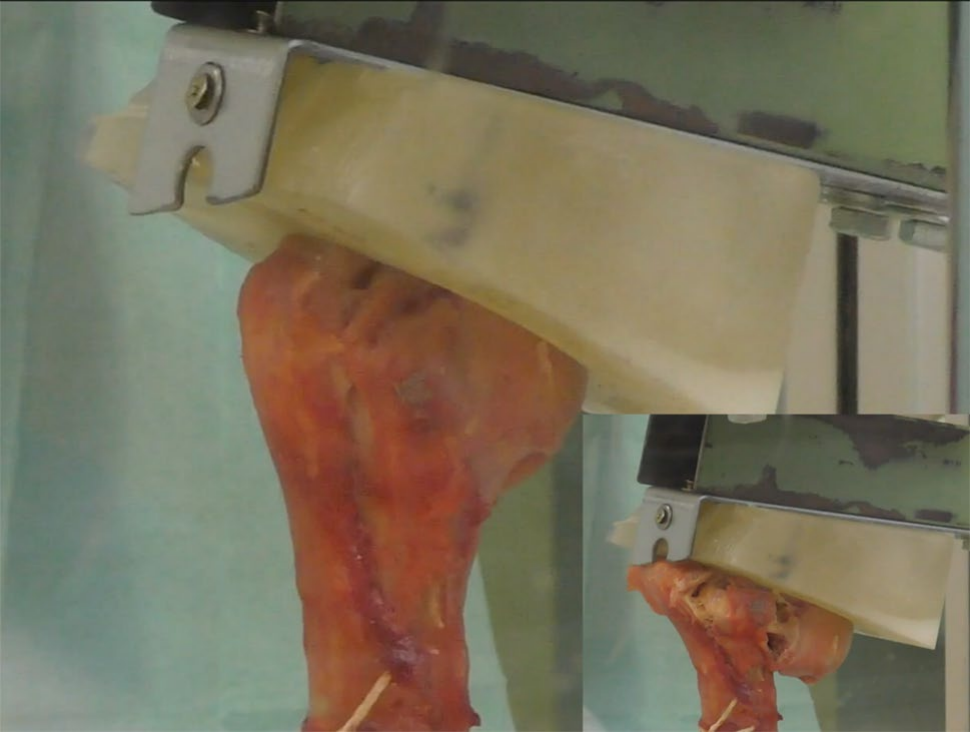
Test set-up simulation of realistic 3-part varus-impacted proximal humeral fractures

A custom drop-test bench with a special tool was designed and fabricated (Fig. 2) to generate the forces required. A comparable system was used in a previous work of the study group [9]. The test bench mainly consists of a steel frame which is mounted on a steel baseplate of 15 mm thickness and 1.5 m in length and width. A special frame was implemented in the test bench to transfer the required kinetic energy on the humeral head.The frame was adjusted belowthe impact stamp. The frame of the test bench includes a height-adjustable crossbeam and an impact beam. Two slide holes within the crossbeam allow passing of two stems to an impact stamp which features two contact plates, one positioned above the crossbeam and one below. An identical manner to achieve proximal humeral fractures was created. When the impact beam is dropped onto the upper contact plate of the impact stamp, the stamp is pushed downwards and deforms the specimen by the amount of overhang of the stamp above the crossbeam. The compression of the specimen is then intended to lead to the desired fracture. Based on prior calculations, an impact velocity of 4 m/s results in up to 210 Joule (J) of kinetic energy induced to the specimen ($E=\frac{1}{2}m{v}^{2};\, E=energy, m=mass, v=velocity)$. Damping mechanisms in addition to the damping effects of the specimens itself are neither needed nor implemented.

The applied kinetic energy for all specimens was 120 Joule.

Following the fracture process, X-ray was conducted to analyze the injury.

All fractures were classified independently by two dedicated trauma surgeons (KW, NO) in compliance with the AO/ASIF recommendations (Fig. 1).

### Surgical procedure

All fractured specimens were anatomically reconstructed and fixed using a 3-hole locking plate (PHILOS; Fa. Synthes; Johnson & Johnson; Norderstedt) in an identical fashion by a single surgeon (ON). Initially no calcar screws were used. The surgical result was reviewed by an X-ray.

**Fig. 3:**
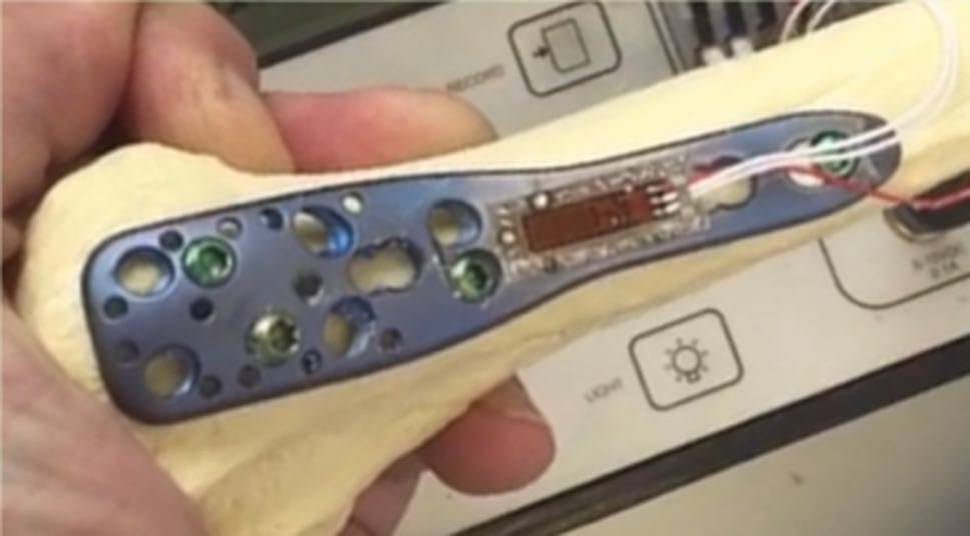
Strain gage (4-wire-350 Ohm; Fa. Vishay) was fixed on locking plates (PHILOS; 3 holes; Fa Depuy/Synthes; Johnson & Johnson)

All 3-holes locking plates were prepared with a strain gage (4-wire-350 Ω; Fa. Vishay) and coated for surface protection (Coat A, Fa. Vishay) (Fig.3).

Prior to the measurements, a calibration of each plate was performed. To keep the measurements errors low, a 4-wire strain was used. The software MCGplus (Fa. HBM) was used to measure the strain and the software CATMAN (Fa. HBM) was used to record the results. The loads applied during the following testing protocol were transferred through the locking plate and resulted in bending forces on the strain gages. In that way, the deformation of the locking plate could be measured by the applied strain gage. The measured strain was used as an estimate for construct stability. A comparable system was applied in a previous work of the study group [10].

### Biomechanical testing

**Fig. 4:**
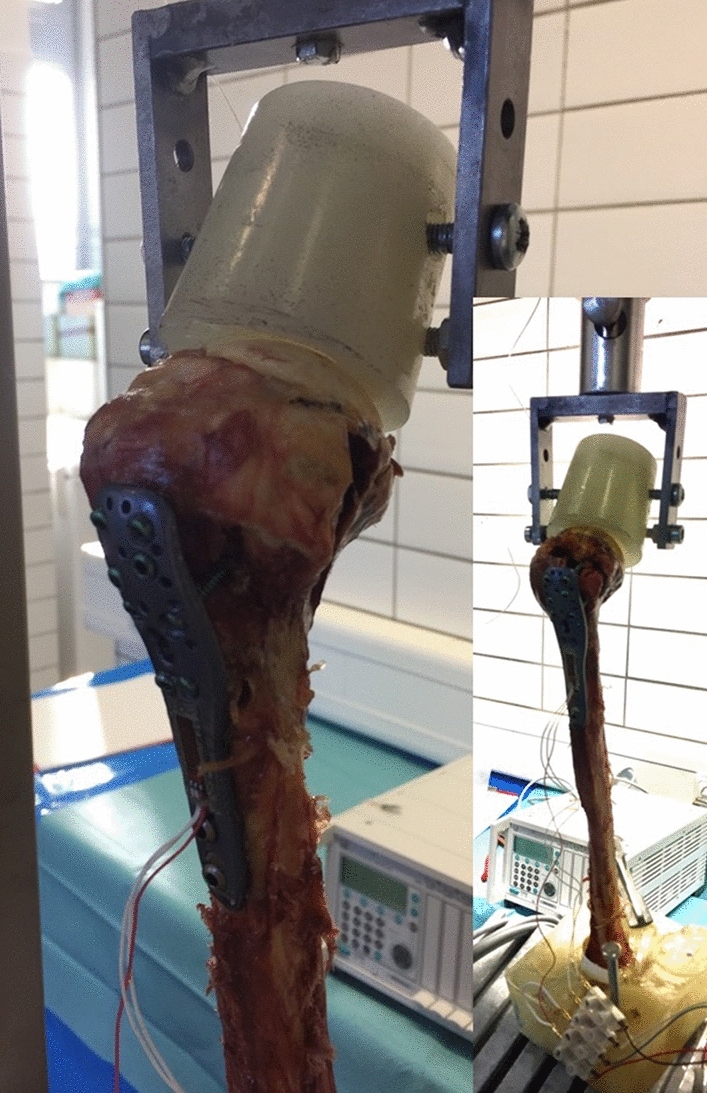
Biomechanical testing set-up (Fa. ZwickRoell, Ulm) with the distal humerus fixaed an performed bowl which is intended to imitate the glenoid and the acromion

The specimens were fixed in an identical setup for direct application of axial loadings in a material testing machine. The distal fragment was fixed. The axial load was applied by a performed bowl which is intended to imitate the glenoid and the acromion. The distal fragment of the humeri was fixed to simulate a realistic load of the shoulder. A constant axial load was induced to test each specimen. The maximum force applied was 200 N. Each specimen was tested without (OC), with two short (2SC) and only 1 long (1LC) as well as 2 long calcar screws (2LC), and strain of the locking plate was measured continuously (Fig. 4).

To verify the study design, one specimen was tested without any fracture (control group). The locking plate was fixed identically, and axial load (100 N, 200 N) was applied in the testing machine. The strain of the locking plate was measured via strain gage.

### Statistical analysis

Statistical analysis was performed using IBM SPSS Statistics. The Kolmogorov–Smirnov test determined normal distribution. Two-sided tests were performed to detect any significant statistical differences. We used descriptive statistics to summarize mean and standard deviations. Correlation analyses to bending force at 200 N and HU were conducted (Pearson ‘s test). The level of significance was defined as a *p* value of < 0.05.

## Results

In all 16 specimens, a realistic impacted 3-part varus fracture was created using the described setup. All fractures were classified without deviation or difference.

**Fig. 5:**
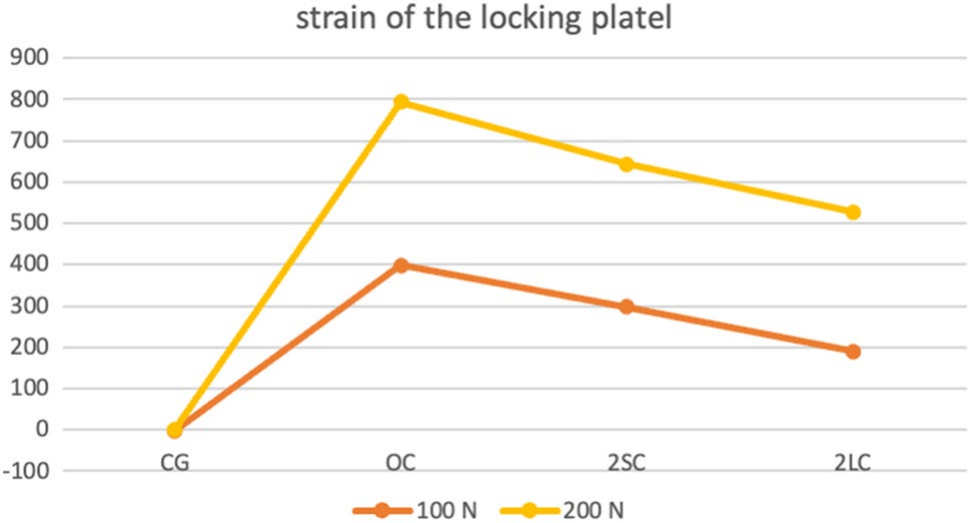
Mean measured strain of the locking plate by axial load (100 N and 200 N), strain was measured in µm/m (y-axis); control group was without a fracture (CG), no calcar screws (OC), with two short calcar screws (2SC) and with two long calcar screws (2LS)

Stresses and strains of the locking plate were measured at 20, 50, 100, 125, 150 and 200 Newton for each group (OC, 2SC, 1LC and 2LC). The available data were summarized in Table 1 and Fig. 5.

**Table 1:** Overview bending force of the locking plate in µm/m; group OC/1LC/2LC/2SC for 20, 50, 70, 100, 125, 150 and 200 Newton

Group	Min	Max	Mean	SD
Control group				
50 N	− 14.1	12.4	− 1.91	8.09
100 N	− 15.1	12	− 2	8.62
150 N	− 14.3	5.4	− 0.99	8.16
200 N	− 12.2	11	1.34	8.62
Without calcar screws (OC)				
50 N	40	341.3	192.98	94.38
100 N	119.8	710.3	398.66	177.48
150 N	256.7	1002.4	583.21	226.64
200 N	396.6	1433.8	793.31	312.29
One long calcar screw (1LC)				
50 N	− 18.2	321.3	143.14	118.96
100 N	− 27.6	634.9	291.39	226.94
150 N	17.2	813.2	435.31	296.59
200 N	76.8	1390.3	643.41	426.24
Two short calcar screws (2SC)				
50 N	23.7	247.5	146.89	80.14
100 N	60.2	540.9	298.19	160.57
150 N	105.3	855.2	448.73	248.25
200 N	162.8	1175.4	619.07	340.58
Two long calcar screws (2LC)				
50 N	− 10.7	174.5	77.84	68.14
100 N	14.9	485.7	190.28	153.24
150 N	45.1	770.2	303.54	236.64
200 N	85.7	1098.3	527.31	338.68

The mean HU of the distal humerus was 247.62 (min. 205; max. 278; SD 28) sagittal and 203.13 (min. 143; max. 246; SD 33). No significant correlation between HU (axial/sagittal) and the bending force was shown (*r* = − 0.643; *p* = 0.168/*r* = − 0.697; *p* = 0.124).

Strain of the plate was significantly higher without any calcar screws installed (804,64 µm/m) than with short (619,07 µm/m; *p* = 0.02) or long calcar screws installed (527,31 µm/m; *p* = 0.007) at maximum load (200 N). Additionally, strain using short calcar screws was noticeably higher in comparison to using long calcar screws (619,07 µm vs. 477,57 µm; *p* = 0.03). A significant difference between groups 2SC and 2LC was detected at each load (Fig. 5). Moreover, a significantly higher strain and stress of the locking plate was observed in group 1LC than in group 2LC, in especially at a load of 175 N (*p* = 0.01).

In the control group, no strain was measured at 100 N nor 200 N (– 2 µm/m; 1,34 µm/m).

### Group OC

The mean strain was 192,98 µm/m (min. 40 µm/m; max. 341,3 µm/m; SD 94,38) at 50 N, 398,66 µm/m (min. 119,8 µm/m max. 710,3 µm/m; SD 177,48) at 100 N, 583,21 µm/m (min. 256,7 µm/m; max. 1002,4 µm/m; SD 226,64) at 150 N and 793,31 µm/m (min. 396,6 µm/m; max. 1433,8 µm/m; SD 312,28) at 200 N.

### Group 2SC

The mean strain at 50 N was 146,87 µm/m (min. 23,7 µm/m; max. 247,5 µm/m; SD 80,13) at 50 N, 298,17 µm/m (min. 60,2 µm/m; max. 540,9 µm/m; SD 160,57) at 100 N, 448,73 µm/m (min. 105,3 µm/m; max. 855,2 µm/m; SD 248,25) at 150 N and 619,07 µm/m (min. 162,8 µm/m; max. 1175,4 µm/m; SD 340,58) at 200 N.

### Group 2LC

The mean strain was 77,83 µm/m (min. − 10,7 µm/m; max. 174,5 µm/m; SD 66,14) at 50 N, 190,27 µm/m (min. 14,9 µm/m; max. 485,7 µm/m; SD 153,24) at 100 N, 303,53 µm/m (min. 45,1 µm/m; max. 770,2 µm/m; SD 236,63) at 150 N and 527,31 µm/m (min. 85,7 µm/m; max. 1098,3 µm/m; SD 338,67) at 200 N.

## Discussion

Based on the present study, two major conclusions can be drawn. First, the use of calcar screws improves the construct stability of proximal humerus fractures. Second, long calcar screws that are positioned as close as possible to the joint provide further primary stability compared to short calcar screws.

Previous studies have focused on the position of the calcar screws and showed that missing the calcar region results in significant reductions in axial stiffness [3, 5, 11–15]. The current findings add to the narrative that medial column support is deciding to provide primary stability of locked plate fixation in proximal humeral fractures. Padegimas et al. have analyzed the position of the calcar screws in a clinical study and observed that missing the calcar proximally by 12 mm led to significantly higher failure rates [14]. Mehta et al. [15] confirmed the results of this clinical study. Their results have shown that missing the calcar proximally decreased the stability while distal placement increased the stiffness. An in vitro biomechanical study by Lescheid et al. supports this by reporting higher axial, torsional and shear stiffness with the restoration of medial cortical contact [16]. In contrast, Katthagen et al. have placed screws across the medial calcar region and have reported no significant difference with the insertion of calcar screws [5]. So, the position of the calcar screws is deciding for the primary stability of locked proximal humeral fractures. Besides the position, several studies have provided the importance of the calcar screws. Zhang et al. detected higher axial and shear stiffness for synthetic humeri with two-part fractures treated with medial support screws compared to those without medial support screws [17]. Moreover, a cadaveric study of three-part fractures by Ponce et al. reports significantly higher mean load to failure and mean energy to failure with the use of calcar screws in PHILOS locking plates during varus collapse tests [18].

Also, insertion of inferomedial screws in PHILOS plate led to significantly lower mean interfragmentary motion and increased the load to failure in humeri with three-part fractures [11]. The results of the present study underline the importance of the calcar screws. Although the role of calcar screws is not a novelty, in contrast to previous studies, the present study provides two benefits. Firstly, the results based on realistic fractured specimen. Neither sawbone nor osteotomized specimen were used. Secondly, strain gage was used to analyze the construct stability. According to recent studies the use of strain gages for analyzing the stability of the locking plates is an established option [10, 19, 20]. A recently published study by our group has analyzed the primary stability of fixations methods for peri-prosthetic fractures of the humerus using this technique [10]. Also, Stoffel et al. showed that bone stability directly correlates with the bending force of a locking plate [20]. Moreover, the group of Seidel developed a telemetric assessment of bone healing using strain gages on an internal fixateur [21]. The use of the strain gages on locking plates allows to analyze the stability of the plate continuously. Thus, we have very consciously decided against calculating a load of failure. The study design was verified by the control group, no strain was measured in case of a non-fractured specimen. The importance of the calcar screws is not a novelty. However, the use of prefractured specimen and strain gage make this in vitro study highly realistic.

There are limitations of this study. First, we have only performed combined bending and axial loading, torsion was not tested. Moreover, load to failure was not reported. In regards to our method, load to failure was not the aim of the present study. Using the strain gage allows to analyze the construct stability continuously. The biological variable was known to play a significant role in results and thus making it difficult to develop correlations and drawing conclusions. X-ray and CT scans were applied to measure Hounsfield Units (HU) before testing the specimen to exclude the ones with any pathology of the relevant region. According to our results, no significant correlation between the HU and the measured bending forces was found. Nevertheless, the variety of the specimens has an influence on the testing methods and their results. Hence, our group was able to produce identical realistic proximal humeral fractures by a single setup. A post-freezing delay of the freshly frozen cadaveric would be noticed and none of the test have a delay of more than 24 h [22]. The specimens were used with repeated bending loads. However, with the expectation that repeated load could creep in the fracture or the implant over time we have started our test setup without calcar screws.

## Conclusion

Using calcar screws significantly improves the stability of locking plates in realistically simulated impacted 3-part varus proximal humeral fractures. Moreover, longer screws that are positioned closely to the joint have a significant advantage compared to short calcar screws.
